# Safety and Feasibility of Gastrectomy in Super Elderly Patients (Aged ≥ 80): A Propensity Score-Matched Analysis

**DOI:** 10.7759/cureus.50443

**Published:** 2023-12-13

**Authors:** Ippei Yamana, Takahisa Fujikawa, Yuichiro Kawamura, Suguru Hasegawa

**Affiliations:** 1 Surgery, Kokura Memorial Hospital, Kitakyushu, JPN; 2 Gastroenterological Surgery, Fukuoka University Hospital, Fukuoka, JPN

**Keywords:** complication, prognosis, gastrectomy, elderly patients, gastric cancer

## Abstract

Introduction: The benefits of gastrectomy in elderly patients with gastric cancer (GC) remain unknown. This study was conducted to evaluate the short- and long-term outcomes of elderly patients with GC (≥ 80 years) who underwent gastrectomy.

Methods: We enrolled 479 patients (Stages I-IV) who underwent gastrectomy with R0-1 resection. The patients were divided into an elderly group (E group; age ≥ 80 years) (n = 115) and a non-elderly group (NE group; age < 80 years) (n = 364). After propensity score matching (PSM) was performed, the short- and long-term outcomes were compared between the groups.

Results: The rate of postoperative complications (Clavien-Dindo classification ≥ IIIa) in the two groups did not differ significantly (p = 0.657). Before PSM, the five-year overall survival (OS, 35.3% vs. 71.7%, p < 0.001) and disease-specific survival (DSS, 56.8% vs. 81.8%, p < 0.001) in the E group were significantly shorter than that in the NE group, respectively. On the other hand, significant differences between the E and NE groups were not shown in either the five-year OS (35.5% vs. 50.8%, p = 0.0985) or the five-year DSS (56.5% vs. 66.9%, p = 0.274) after PSM.

Conclusion: Gastrectomy for elderly patients with GC can be considered safe based on short-term outcomes. In terms of long-term results, elderly patients are not inferior to non-elderly patients if the patients' backgrounds are the same. On the other hand, the long-term outcomes of elderly GC patients who have various comorbidities are not satisfactory, so we should carefully consider the indications for gastrectomy.

## Introduction

In recent years, the number of gastrointestinal cancer (GC) surgery cases among the elderly has been increasing owing to advances in medical science and the aging of the population [1]. GC is the fourth most common cancer in the world and the second leading cause of cancer-related deaths [2], and the number of reports concerning elderly GC patients has been increasing. Although there are many reports on the clinicopathological characteristics, postoperative complications, and surgical safety of elderly patients with GC [3-5], there are few reports on the long-term prognosis.

Previous studies have shown that the safety of gastrectomy in elderly patients is similar to that in non-elderly patients [3-5]. However, surgeons should carefully consider the indications for gastrectomy in elderly GC patients because of their various comorbidities. In addition, elderly patients over 80 years of age may die of other illnesses despite undergoing radical surgery for GC.

In this study, we evaluated the short- and long-term outcomes of GC patients who underwent gastrectomy at over 80 years of age and evaluated the benefits of surgery for gastric cancer.

## Materials and methods

Patients

After permission from Kokura Memorial Hospital's institutional review board (#22072801), potentially relevant cases were searched from the single institution's prospectively collected surgery database. Following the exclusion of cases of residual gastric cancer, double cancer, gastric tube cancer, and R2 resection, the current study included 479 consecutive patients with pathological Stage I-IV GC who underwent gastrectomy with R0-1 resection between 2012 and 2022 at Kokura Memorial Hospital, Fukuoka, Japan.

Data collection

**Figure 1:**
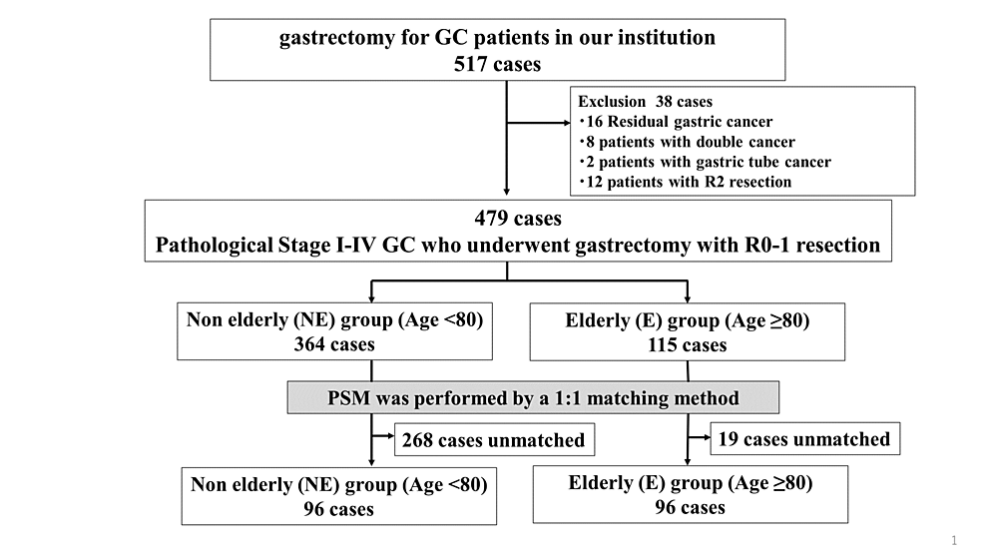
Consort diagram in the current study. GC: gastric cancer; PSM: propensity score-matching.

The patients were divided into an elderly group (n = 115) (E group; age ≥ 80 years) and a non-elderly group (n = 364) (NE group; age < 80 years). Based on preoperative clinical characteristics and pathological stage, propensity score matching (PSM) was carried out. Consequently, the patients were divided into an E group (n = 96) and an NE group (n = 96) (Figure 1). A comparison was made between these groups for the background characteristics, perioperative variables, surgical results, and prognosis of the included patients.

The Eastern Cooperative Oncology Group Scale of Performance Status (PS) was used to report the patients' functional status and symptoms [6]. TNM (tumor (T), nodes (N), and metastases (M)) clinical stage was developed using the TNM classification eighth edition. Prognostic nutritional index (PNI) and geriatric nutritional risk index (GNRI) were used for nutritional assessment. PNI was computed as follows: PNI = serum albumin (g/L) + 5 × total lymphocyte count (109/L) [7]. The formula used to compute the GNRI was GNRI = (14.89 × serum albumin level (g/dL)) + (41.7 × actual body weight/ideal body weight) [8]. Data obtained at the time of admission was used to determine each patient's real body weight and serum albumin level.

For the management of patients receiving antithrombotic therapy (ATT; antiplatelet therapy (APT) and/or anticoagulation therapy (ACT)), we applied our perioperative antithrombotic management protocol (“Kokura" protocol) [9,10]. In brief, a single aspirin was given the day before surgery for antiplatelet-received patients, the anticoagulant drug (warfarin) was switched to heparin or direct oral anticoagulation (DOAC) three to five days before surgery, and DOACs were given up to the day of the surgery. The postoperative thromboembolic risk was evaluated and categorized by the CHADS2 score, which is a cumulative score based on six clinical features: congestive heart failure, hypertension, diabetes mellitus, age ≥75 years (1 point each), and a history of stroke or transient ischemic attack (2 points) [11].

The primary outcomes included five-year overall survival (OS) and disease-specific survival (DSS). The secondary outcomes included postoperative complications. OS was defined as the time from the date of surgery until death from any cause, including death from another disease. DSS was defined as the time from the date of surgery until death from the specific disease. The postoperative complications were defined as the Clavien-Dindo classification (CDC) [12] grade ≥ IIIa complications that occurred within 30 days of surgery.

Surgical procedure and perioperative management

The tumor status and degree of lymph node dissection were determined according to the Japanese Gastric Cancer Treatment Guidelines [13]. Gastrectomy was performed with D2 lymph node dissection for advanced cancer and D1+ lymph node dissection for early gastric cancer, according to the Japanese Gastric Cancer Treatment Guidelines [13]. However, surgeons could decide to refrain from performing lymph node dissection in elderly or high-risk patients. Adjuvant chemotherapy is indicated for patients with pathological stages II and III disease, excluding T3N0. Adjuvant chemotherapy was based on oral 5-fluorouracil derivatives, and the indications for adjuvant chemotherapy depended on the patients’ functional status, including preserved organ function, performance status of 0 or 1, and adequate oral intake. Patients were periodically checked for recurrence via physical examination and blood tests every three months after discharge from the hospital. CT was performed at least every six months after surgery to examine recurrence patterns and survival status.

Statistical analysis

Continuous variables were expressed as medians with ranges. The propensity score was calculated using a logistic regression model. Preoperative variables were entered into PSM based on preoperative clinical parameters, including gender, performance status, pathological stage, and CHADS2 score. PSM was performed using a 1:1 matching method. Statistical comparisons for categorized data were made using the chi-squared test or Fisher's exact probability test, and the Student's t-test was used to analyze differences for continuous values. All P-values were two-sided and P-values less than .05 were considered statistically significant. For the survival analysis, Kaplan-Meier survival analysis was performed as the univariate analysis. All statistical analyses were performed with EZR (Saitama Medical Centre, Jichi Medical University, Shimotsuke, Japan), which is a graphical user interface for R (The R Foundation for Statistical Computing, Vienna, Austria, version 2.13.0) [14].

## Results

Search results and study characteristics

The characteristics of the patients in the E and NE groups before and after PSM are shown in Tables 1-3. All the following parameters showed significant differences between the NE and E groups before PSM: performance status 2 or higher (4.9% vs. 29.6%; p < 0.001), history of congestive heart failure (10.4% vs. 27.8%; p < 0.001), history of PCI (14.6% vs. 27.8%; p = 0.001), CHADS2 score 2 or higher (32.7% vs. 73.9%; p<0.001), APT (28.6% vs. 43.5%; p = 0.004), PNI ≥ 40 (89.8% vs. 78.3%; p = 0.02), GNRI ≥ 90 (88.7% vs. 72.2%; p < 0.001), pathological stage (p < 0.001), operating time (p = 0.03), and the days of postoperative hospital stay (p < 0.001). Using PSM, the patient backgrounds were no longer different between the E and NE groups.

**Table 1:** Background characteristics of patients before and after propensity score matching. ^#^Values indicate statistical significance. PSM: propensity score matching; APT: antiplatelet therapy; ACT, anticoagulant therapy; PNI, prognostic nutritional index; GNRI, geriatric nutritional risk index; CABG, coronary artery bypass graft surgery; PCI, percutaneous coronary intervention; CHADS2 score, congestive heart failure, hypertension, age ≥75 years, and diabetes mellitus, each given 1 point; and a past history of transient ischemic attack or stroke given 2 points; NE group, non-elderly group; E group, elderly group

Factor	Group	NE group, before PSM (n=364)	E group, before PSM (n=115)	p-value	NE group, after PSM (n=96)	E group, after PSM (n=96)	p-value
Gender (%)	Male	275 (75.5)	83 (72.2)	0.463	75 (78.1)	73 (76.0)	0.864
	Female	89 (24.5)	32 (27.8)		21 (21.9)	23 (24.0)	
Age, median (range)		70(34-79)	83(80-94)	<0.001^#^	73 (48-79)	83(80-94)	<0.001^#^
Performance status (%)	0-1	346 (95.1)	81 (70.4)	<0.001^#^	83 (86.5)	80 (83.3)	0.687
	2-4	18 (4.9)	34 (29.6)		13 (13.5)	16 (16.7)	
Cerebral infarction (%)		28 (7.7)	14 (12.2)	0.184	18 (18.8)	11 (11.5)	0.226
Dialysis (%)		11 (3.0)	2 (1.7)	0.742	4 (4.2)	2 (2.1)	0.683
History of CABG (%)		12 (3.3)	5 (4.3)	0.57	4 (4.2)	4 (4.2)	1
History of congestive heart failure (%)		38 (10.4)	32 (27.8)	<0.001^#^	21 (21.9)	23 (24.0)	0.864
History of PCI (%)		53 (14.6)	33 (28.7)	0.001^#^	18 (18.8)	29 (30.2)	0.093
CHADS_2_ score (%)	0-1	245 (67.3)	30 (26.1)	<0.001^#^	27 (28.1)	30 (31.2)	0.752
	2-6	119 (32.7)	85 (73.9)		69 (71.9)	66 (68.8)	
APT (%)		104 (28.6)	50 (43.5)	0.004^#^	38 (39.6)	43 (44.8)	0.559
ACT (%)		29 (8.0)	15 (13.0)	0.136	16 (16.7)	11 (11.5)	0.407
PNI ≥40 (%)		327 (89.8)	90 (78.3)	0.002^#^	15 (15.6)	14 (14.6)	1
GNRI ≥90 (%)		323 (88.7)	83 (72.2)	<0.001^#^	83 (85.5%)	72 (75%)	0.066

**Table 2:** Operative procedures and surgical outcome before and after propensity score matching. ^#^Values indicate statistical significance. PSM, propensity score matching; Laparo, laparoscopic; NE group, non-elderly group; E group, elderly group

Factor	Group	NE group, before PSM (n=364)	E group, before PSM (n=115)	p-value	NE group, after PSM (n=96)	E group, after PSM (n=96)	p-value
Operating method (%)	Open	126 (34.6)	49 (42.6)	0.306	35 (36.5)	45 (46.9)	0.33
	Laparo	199 (54.7)	56 (48.7)		52 (54.2)	42 (43.8)	
	Robot	39 (10.7)	10 (8.7)		9 (9.4)	9 (9.4)	
Resection range (%)	Total	107 (29.4)	39 (33.9)	0.621	30 (31.2)	36 (37.5)	0.512
	Distal	247 (67.9)	74 (64.3)		62 (64.6)	58 (60.4)	
	Proximal	10 (2.7)	2 (1.7)		4 (4.2)	2 (2.1)	
Operating time (min), median (range)		312 (128-648)	273 (84-657)	0.03^#^	309 (152-632)	273 (84-657)	0.038^#^
Blood loss (ml), median (range)		42 (0-2550)	70 (0-340)	0.056	75 (0-2440)	87.5 (0-3400)	0.37
Length of postoperative hospital stay (d), median (range)		14 (3-143)	15 (8-112)	<0.001^#^	15 (7-143)	15 (8-112)	0.362

**Table 3:** Oncological outcome before and after propensity score matching. ^#^Values indicate statistical significance. PSM, propensity score matching; NE group, non-elderly group; E group, elderly group

Factor	Group	NE group, before PSM (n=364)	E group, before PSM (n=115)	p-value	NE group, after PSM (n=96)	E group, after PSM (n=96)	p-value
T factor (%)	1	189 (51.9)	37 (32.2)	0.001^#^	38 (39.6)	35 (36.5)	0.827
	2	50 (13.7)	16 (13.9)		15 (15.6)	12 (12.5)	
	3	76 (20.9)	34 (29.6)		23 (24.0)	27 (28.1)	
	4	49 (13.5)	28 (24.3)		20 (20.8)	22 (22.9)	
N factor (%)	0	225 (61.8)	53 (46.1)	0.001^#^	37 (38.5)	46 (47.9)	0.467
	1	67 (18.4)	18 (15.7)		20 (20.8)	15 (15.6)	
	2	28 (7.7)	20 (17.4)		12 (12.5)	14 (14.6)	
	3	44 (12.1)	24 (20.9)		27 (28.1)	21 (21.9)	
M factor (%)	0	353 (97.0)	110 (95.7)	0.551	90 (93.8)	93 (96.9)	0.497
	1	11 (3.0)	5 (4.3)		6 (6.2)	3 (3.1)	
Number of lymph nodes metastasis, median (range)		0 (0-49)	1 (0-58)	0.001^#^	1 (0-39)	1 (0-58)	0.74
Pathological stage (%)	1	204 (56.0)	44 (38.3)	0.001^#^	39 (40.6)	40 (41.7)	1
	2	90 (24.7)	28 (24.3)		22 (22.9)	21 (21.9)	
	3	60 (16.5)	37 (32.2)		30 (31.2)	31 (32.3)	
	4	10 (2.7)	6 (5.2)		5 (5.2)	4 (4.2)	
R0/1	0	350 (96.2)	108 (93.9)	0.303	90 (93.8)	89 (92.7)	1
	1	14 (3.8)	7 (6.1)		6 (6.2)	7 (7.3)	

Postoperative morbidity

Postoperative complications (CDC score ≥ IIIa) before and after PSM are shown in Table 4. Both before and after PSM, the rate of postoperative complications (CDC ≥ IIIa) in the E and NE groups did not differ significantly (p = 0.435 and p = 0.657, respectively). Before PSM, the rates of anastomotic leakage, acute heart failure, pneumonia, and anastomotic stenosis in the E group were significantly higher than those in the NE group (p = 0.0401, p = 0.0317, p = 0.0136, and p = 0.0136, respectively). After PSM, the postoperative complication rates were not statistically different. Before PSM, three patients (0.6%) died within 30 days of surgery. One patient had a postoperative myocardial infarction and died of heart failure on postoperative day three. One patient developed pneumonia postoperatively and died on postoperative day 20. Another patient had postoperative adrenal insufficiency and died on postoperative day 26.

**Table 4:** The patients’ postoperative morbidity before and after propensity score matching. ^#^Values indicate statistical significance. PSM, propensity score matching; CDC, Clavien-Dindo classification; NE group, non-elderly group; E group, elderly group

Factor	Group	NE group, before PSM (n=364)	E group, before PSM (n=115)	p-value	NE group, after PSM (n=96)	E group, after PSM (n=96)	p-value
CDC over IIIa (%)		27 (7.4)	11 (9.6)	0.435	13 (13.5)	10 (10.4)	0.657
Bleeding		2	8	1	3	3	1
Anastomotic leakage		4	5	0.0401^#^	2	4	0.683
Acute heart failure		2	4	0.0317^#^	2	2	1
Pneumonia		0	3	0.0136^#^	0	2	0.497
Anastomotic stenosis		0	3	0.0136^#^	0	2	0.497
Cerebral infarction		2	0	1	2	0	0.497
Pancreatic fistula		０	2	0.0573	0	0	1
Cholangitis		1	1	0.423	1	0	1
Adrenal insufficiency		0	1	0.24	0	0	1

Survival analyses before and after PSM

**Figure 2:**
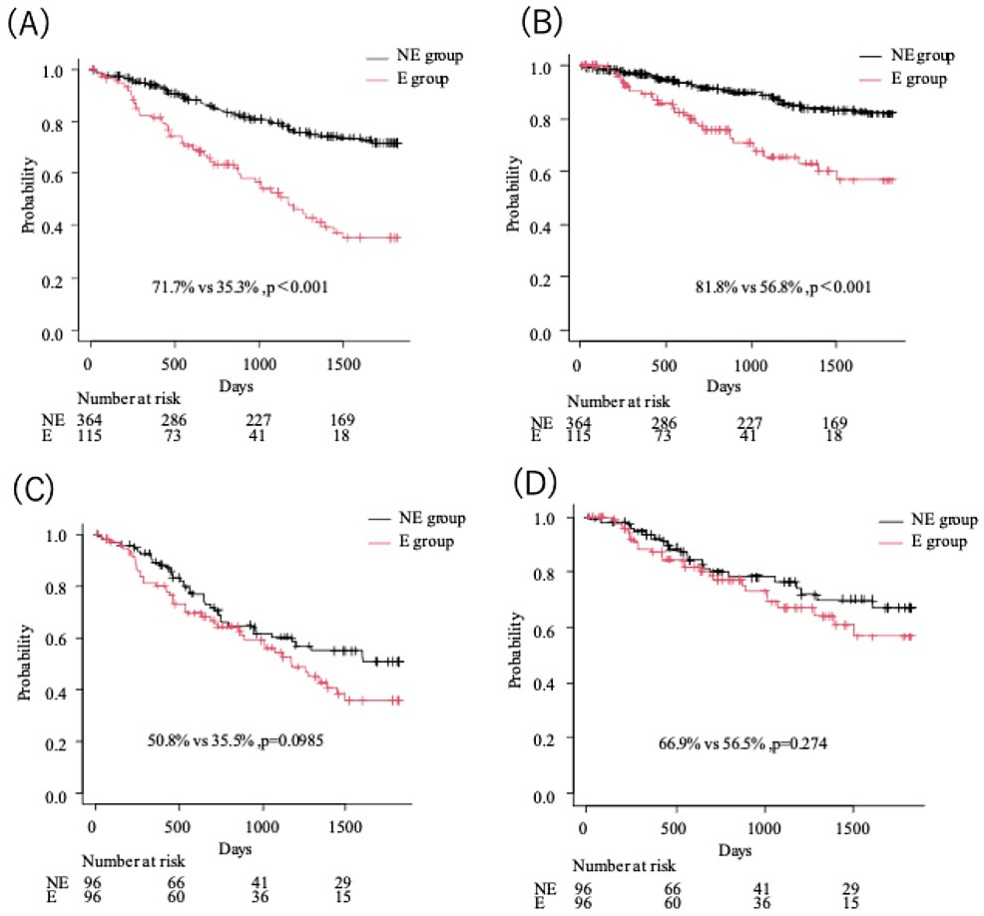
The five-year overall survival (OS) and disease-specific survival (DSS) in the NE and E groups before and after propensity score matching (PSM). (A, B) Before PSM, the five-year OS (Figure 2A, 35.3 % vs. 71.7 %, p<0.001) and DSS (Figure 2B, 56.8 % vs. 81.8 %, p<0.001) in the E group were significantly shorter than that in the NE group, respectively. (C, D) Significant differences between the E and NE groups were shown in neither the 5-year OS (Figure 2C, 35.5% vs. 50.8%, p = 0.0985) nor DSS (Figure 2D, 56.5% vs. 66.9%, p = 0.274) after PSM. NE group, non-elderly group; E group, elderly group

Figure 2 shows the five-year OS and DSS in the NE and E groups before and after PSM. The five-year OS in the E group was significantly shorter than that in the NE group (35.3% vs. 71.7%, p<0.001) before PSM (Figure 2A). The five-year DSS in the E group was also significantly shorter than that in the NE group (56.8% vs. 81.8%, p<0.001) before PSM (Figure 2B). On the other hand, the five-year OS in the E group was not significantly different between the E and NE groups (35.5% vs. 50.8%, p = 0.0985) after PSM (Figure 2C), nor was the five-year DSS (56.5% vs. 66.9%, p = 0.274) after PSM (Figure 2D).

## Discussion

In Japan, 20% of patients with GC are over 80 years of age [15]. In our institution, 24% of patients with GC who underwent gastrectomy were over 80 years of age. According to the Japanese Ministry of Health Labour and Welfare, the average life expectancy in 2010 was 79.64 years for men and 86.39 years for women. The average remaining life expectancy at 80 years of age was reported to be 6.18 years for men and 8.30 years for women. Based on these outcomes, gastrectomy for patients with GC over 80 years of age is considered worthwhile. However, elderly patients have various comorbidities and a poorer nutritional status than younger patients, which puts them at risk for postoperative complications. Our results showed that although elderly patients had significantly lower performance status and poorer nutritional status than non-elderly patients, the rates of postoperative complications (CDC ≥ IIIa) in the E and NE groups did not differ significantly. Moreover, elderly patients were not inferior to non-elderly patients in terms of long-term outcomes if the patients’ backgrounds were comparable.

Hikage et al. [16] suggested that the overall postoperative complication rates of the very elderly group (age ≥ 85 years) and the elderly group (age 75-84 years) were comparable. Yang et al. [17] also suggested that the rates of overall and severe (grade ≥ IIIa) complications in the E (age ≥ 80 years) and NE groups were comparable. In our study, the rates of postoperative complications (CDC ≥ IIIa) in the E and NE groups did not differ significantly before and after PSM. However, before PSM, the rates of anastomotic leakage, acute heart failure, anastomotic stenosis, and pneumonia were significantly higher in the E group than in the NE group (p = 0.0401, 0.0317, 0.0136, and 0.0136, respectively). After PSM, these factors were not significantly different (p= 0.683, 1.0, 0.497, and 0.497, respectively). All comorbidities being equivalent, these findings suggest that elderly patients can undergo gastrectomy as safely as non-elderly patients.

Several studies have reported the prognosis of postoperative gastric cancer in the elderly. Matsunaga et al. [18] reported that five-year OS was significantly lower in the E group (age ≥ 75 years) than in the NE group (age < 75 years) in Stage I-III (E group vs. NE group; Stage I: 80.1% vs. 88.3%, p = 0.034, Stage II: 64.4% vs. 80%, p = 0.032, Stage III: 18.3% vs. 44.3%, p = 0.003), and five-year DSS was significantly lower in the E group (age ≥ 75 years) than in the NE group (age < 75 years) in stage III (E group vs. NE group; 23% vs. 59.4%, p = 0.004). Hashimoto et al. [19] suggested that the DSS was similar between the elderly group (age ≥ 75 years) and the non-elderly group (age < 75 years) (p = 0.743), whereas the OS in the elderly group was significantly shorter than that in the non-elderly group (p < 0.001) because of a higher incidence of death from other diseases throughout all gastric cancer stages. In the present study, to minimize bias due to patient characteristics between the E and NE groups, PSM was performed. Before PSM, the five-year OS in the E group was significantly shorter than that in the NE group (35.3% vs. 71.7%, p < 0.001). The five-year DSS in the E group was also significantly shorter than that in the NE group (56.8% vs. 81.8%, p < 0.001). However, after PSM, the five-year OS in the E group was not significantly different from that in the NE group (35.5% vs. 50.8%, p = 0.0985), and the same was true for the five-year DSS (56.5% vs. 66.9%, p = 0.274). These results indicate that the prognosis is worse for E patients with comorbidities than for NE patients. However, the prognosis would be similar, regardless of age, if the backgrounds were comparable.

Hirahara et al. [20] reported that multivariate analysis demonstrated that the American Society of Anesthesiologists (ASA) score, performance status, tumor differentiation, carcinoembryonic antigen (CEA), and GNRI independently predicted OS in adults aged > 65 years. Furuke et al. [21] suggested that a low GNRI is an independent prognostic factor for poor OS at all ages. In our results, significant differences were observed between the NE and E groups in PNI or GNRI. If a patient is found to have a poor nutritional status preoperatively, aggressive nutritional support should be performed. Furthermore, endoscopic submucosal dissection or follow-up observation without surgery may also be an option for patients with poor nutritional status.

The present study has some limitations. First, it was a retrospective analysis performed at a single institution. Second, in our institution, patients undergoing ATT account for approximately 40% of patients undergoing gastrointestinal surgery, and the rate of postoperative bleeding is relatively high. In this study, the number of GC patients receiving ATT was 174 out of 479 cases (36.3%), and the number of cases of postoperative bleeding was 10 (2.1%). Furthermore, six out of 10 cases received ATT. Therefore, the results in the current study need to be confirmed in another cohort or in a prospective multicenter study.

## Conclusions

We evaluated the short- and long-term outcomes of gastrectomy with elderly GC patients (over 80). In terms of short-term outcomes, the incidence of postoperative complications (CDC score ≥IIIa) is comparable between elderly and non-elderly patients. The long-term outcomes of elderly patients are not inferior to those of non-elderly patients, provided patients have similar backgrounds. However, the long-term outcomes of elderly GC patients who have cardiovascular event risk and lower nutritional status are not satisfactory. Based on these findings, we suggest that it is most important to carefully select elderly GC patients for gastrectomy.

## References

[1] Bray F, Jemal A, Grey N, Ferlay J, Forman D (2012). Global cancer transitions according to the Human Development Index (2008-2030): a population-based study. Lancet Oncol.

[2] Ferlay J, Soerjomataram I, Dikshit R (2015). Cancer incidence and mortality worldwide: sources, methods and major patterns in GLOBOCAN 2012. Int J Cancer.

[3] Aoyama T, Maezawa Y, Yoshikawa T (2019). Comparison of weight and body composition after gastrectomy between elderly and non-elderly patients with gastric cancer. In Vivo.

[4] Pucheanu X, Beuran M (2015). Bleeding gastric cancer in young and elderly patients. J Med Life.

[5] Lim JH, Lee DH, Shin CM, Kim N, Park YS, Jung HC, Song IS (2014). Clinicopathological features and surgical safety of gastric cancer in elderly patients. J Korean Med Sci.

[6] Sørensen JB, Klee M, Palshof T, Hansen HH (1993). Performance status assessment in cancer patients. An inter-observer variability study. Br J Cancer.

[7] Buzby GP, Mullen JL, Matthews DC, Hobbs CL, Rosato EF (1980). Prognostic nutritional index in gastrointestinal surgery. Am J Surg.

[8] Bouillanne O, Morineau G, Dupont C (2005). Geriatric Nutritional Risk Index: a new index for evaluating at-risk elderly medical patients. Am J Clin Nutr.

[9] Fujikawa T, Tanaka A, Abe T, Yoshimoto Y, Tada S, Maekawa H (2015). Effect of antiplatelet therapy on patients undergoing gastroenterological surgery: thromboembolic risks versus bleeding risks during its perioperative withdrawal. World J Surg.

[10] Fujikawa T, Tanaka A, Abe T, Yoshimoto Y, Tada S, Maekawa H, Shimoike N (2013). Does antiplatelet therapy affect outcomes of patients receiving abdominal laparoscopic surgery? Lessons from more than 1,000 laparoscopic operations in a single tertiary referral hospital. J Am Coll Surg.

[11] Gage BF, Waterman AD, Shannon W, Boechler M, Rich MW, Radford MJ (2001). Validation of clinical classification schemes for predicting stroke: results from the National Registry of Atrial Fibrillation. JAMA.

[12] Dindo D, Demartines N, Clavien PA (2004). Classification of surgical complications: a new proposal with evaluation in a cohort of 6336 patients and results of a survey. Ann Surg.

[13] (2023). Japanese Gastric Cancer Treatment Guidelines 2021 (6th edition). Gastric Cancer.

[14] Kanda Y (2013). Investigation of the freely available easy-to-use software 'EZR' for medical statistics. Bone Marrow Transplant.

[15] Matsuda T, Marugame T, Kamo K, Katanoda K, Ajiki W, Sobue T (2011). Cancer incidence and incidence rates in Japan in 2005: based on data from 12 population-based cancer registries in the Monitoring of Cancer Incidence in Japan (MCIJ) project. Jpn J Clin Oncol.

[16] Hikage M, Tokunaga M, Makuuchi R (2018). Surgical outcomes after gastrectomy in very elderly patients with gastric cancer. Surg Today.

[17] Yang XW, Zhu SH, Li PZ, Li WZ, Sun XL (2018). Outcomes of laparoscopic gastrectomy for gastric cancer in elderly patients. J BUON.

[18] Matsunaga T, Ishiguro R, Miyauchi W (2021). Appraisal of long-time outcomes after curative surgery in elderly patients with gastric cancer: a propensity score matching analysis. BMC Surg.

[19] Hashimoto T, Kurokawa Y, Mikami J (2019). Postoperative long-term outcomes in elderly patients with gastric cancer and risk factors for death from other diseases. World J Surg.

[20] Hirahara N, Matsubara T, Fujii Y (2020). Preoperative geriatric nutritional risk index is a useful prognostic indicator in elderly patients with gastric cancer. Oncotarget.

[21] Furuke H, Matsubara D, Kubota T (2021). Geriatric nutritional risk index predicts poor prognosis of patients after curative surgery for gastric cancer. Cancer Diagn Progn.

